# Complete Circular Genome Sequence of Successful ST8/SCC*mec*IV Community-Associated Methicillin-Resistant *Staphylococcus aureus* (OC8) in Russia: One-Megabase Genomic Inversion, IS*256*’s Spread, and Evolution of Russia ST8-IV

**DOI:** 10.1371/journal.pone.0164168

**Published:** 2016-10-14

**Authors:** Tsai-Wen Wan, Olga E. Khokhlova, Yasuhisa Iwao, Wataru Higuchi, Wei-Chun Hung, Ivan V. Reva, Olga A. Singur, Vladimir V. Gostev, Sergey V. Sidorenko, Olga V. Peryanova, Alla B. Salmina, Galina V. Reva, Lee-Jene Teng, Tatsuo Yamamoto

**Affiliations:** 1 Department of Epidemiology, Genomics, and Evolution, International Medical Education and Research Center, Niigata, Japan; 2 Department of Clinical Laboratory Sciences and Medical Biotechnology, National Taiwan University College of Medicine, Taipei, Taiwan; 3 Russia-Japan Center of Microbiology, Metagenomics and Infectious Diseases, Krasnoyarsk State Medical University named after Professor V.F. Vojno-Yasenetsky, Krasnoyarsk, Russia; 4 Department of Microbiology, Krasnoyarsk State Medical University named after Professor V.F. Vojno-Yasenetsky, Krasnoyarsk, Russia; 5 Division of Bacteriology, Department of Infectious Disease Control and International Medicine, Niigata University Graduate School of Medical and Dental Sciences, Niigata, Japan; 6 Department of Microbiology and Immunology, Kaohsiung Medical University, Kaohsiung, Taiwan; 7 Department of Clinical and Fundamental Medicine, Far Eastern Federal University School of Biomedicine, Vladivostok, Russia; 8 Department of Venereal and Skin Diseases and Cosmetology, Pacific State Medical University, Vladivostok, Russia; 9 Department of Medical Microbiology and Molecular Epidemiology, Scientific Research Institute of Children’s Infections, St. Petersburg, Russia; 10 Research Institute of Molecular Medicine and Pathobiochemistry, Krasnoyarsk State Medical University named after Professor V.F. Vojno-Yasenetsky, Krasnoyarsk, Russia; University of California San Francisco, UNITED STATES

## Abstract

ST8/SCC*mec*IV community-associated methicillin-resistant *Staphylococcus aureus* (CA-MRSA) has been a common threat, with large USA300 epidemics in the United States. The global geographical structure of ST8/SCC*mec*IV has not yet been fully elucidated. We herein determined the complete circular genome sequence of ST8/SCC*mec*IVc strain OC8 from Siberian Russia. We found that 36.0% of the genome was inverted relative to USA300. Two IS*256*, oppositely oriented, at IS*256*-enriched hot spots were implicated with the one-megabase genomic inversion (MbIN) and vSaβ split. The behavior of IS*256* was flexible: its insertion site (*att*) sequences on the genome and junction sequences of extrachromosomal circular DNA were all divergent, albeit with fixed sizes. A similar multi-IS*256* system was detected, even in prevalent ST239 healthcare-associated MRSA in Russia, suggesting IS*256*’s strong transmission potential and advantage in evolution. Regarding epidemiology, all ST8/SCC*mec*IVc strains from European, Siberian, and Far Eastern Russia, examined had MbIN, and geographical expansion accompanied divergent *spa* types and resistance to fluoroquinolones, chloramphenicol, and often rifampicin. Russia ST8/SCC*mec*IVc has been associated with life-threatening infections such as pneumonia and sepsis in both community and hospital settings. Regarding virulence, the OC8 genome carried a series of toxin and immune evasion genes, a truncated giant surface protein gene, and IS*256* insertion adjacent to a pan-regulatory gene. These results suggest that unique single ST8/*spa*1(t008)/SCC*mec*IVc CA-MRSA (clade, Russia ST8-IVc) emerged in Russia, and this was followed by large geographical expansion, with MbIN as an epidemiological marker, and fluoroquinolone resistance, multiple virulence factors, and possibly a multi-IS*256* system as selective advantages.

## Introduction

Community-associated methicillin-resistant *Staphylococcus aureus* (CA-MRSA) is a class of MRSA, that has been reported since the 1990s [1–4]. CA-MRSA is primarily isolated in the community [2], but is also isolated in hospital settings [5]. CA-MRSA is generally associated with skin soft tissue infections (SSTI), but also life-threatening, severe, and invasive infections such as pneumonia (including necrotizing cases), sepsis, bloodstream infections, osteomyelitis, and lung (as well as pelvic and epidural) abscesses [1,3,4,6–8]. Large outbreaks of CA-MRSA include the USA300 epidemic with serious invasive infections in the United States in 2007 [2,4,9,10].

CA-MRSA exhibits heterogeneous genetic backgrounds, regarding multilocus sequence types (ST types), protein A gene (*spa*) types, or staphylococcal cassette chromosome *mec* (SCC*mec*) types [4,9,11–14]. The most characterized successful CA-MRSA include the ST8 lineage, such as ST8/SCC*mec*IV (USA300) [4,9,10–12,14,15], and also the lineages of ST30/SCC*mec*IV [3,4,8,16–18], ST59/SCC*mec*V or IV [4,8,19–24], and ST80/SCC*mec*IV [3,4,16,25,26]: each lineage includes diverse *spa* types.

MRSA achieves its dynamic evolution mainly through the action of mobile genetic elements, such as insertion sequences and transposons, plasmids, phages, and *S*. *aureus* pathogenicity islands (SaPIs), and also through mutations [4,10,13,14,27–31]. Successful CA-MRSA may have each characteristic genetic trait (or a combination) for virulence and/or drug resistance [4,10,13,14]: for example, USA300 has the Panton-Valentine leukocidin (PVL)-encoding phage and the two-cassette array of SCC*mec*IVa and the arginine catabolic mobile element (ACME) [15], and has become multidrug-resistant including resistance to fluoroquinolones [11,32]; and ST59/SCC*mec*V MRSA form Taiwan has the PVL-converting phage and the mobile element structure with IS*1216V* (MES_PM1_) encoding for multidrug resistance [22,23].

Regarding insertion sequences, IS*256* was originally found in *S*. *aureus* as the terminal inverted repeat (IR) of transposon Tn*4001*, encoding for resistance to aminoglycosides (such as gentamicin) [33]. IS*256* exists as multiple copies in a cell [33] with a preferred insertion site [34]. IS*256* may affect virulence and drug resistance gene expression [30,34], and may also serve as a crossover point for homologous recombination [33]. IS*256* is not common among *S*. *aureus*; for example, USA300 has no IS*256* [30].

CA-MRSA possesses common bacteriological features, such as the elevated expression of cytolytic peptides (phenol-soluble modulins, PSMs, or δ-hemolysin, Hld) [9,35], less multidrug resistance [8,36], and low minimum inhibitory concentrations (MICs) for oxacillin and imipenem [36]. Moreover, CA-MRSA exhibits SCC*mec* type IV or V in many cases [4,8,13,14,37], and often produces PVL and carries ACME [4,8,14,15,37]. The ST types of globally distributed CA-MRSA include, for example, ST8, ST30, ST59, and ST80, as described above.

In Russia, Sidorenko’s group investigated the molecular characteristics (such as ST/*spa*/SCC*mec* types, drug resistance, and virulence genes) of MRSA obtained in a nation wide hospital MRSA surveillance. The prevalence of MRSA in Russia varies from 0 to 80% [38]. The prevalent CA-MRSA lineage exhibits ST8/SCC*mec*IVc, with *spa* types such as *spa*1(t008) and 363(t024) [38]. Prevalent healthcare-associated MRSA (HA-MRSA [2]) is the ST239/SCC*mec*III lineage, with *spa* types such as *spa*3(t037) [38], which is one of the most globally distributed HA-MRSA lineages [39].

Our international (Japan-Russia, more recently Japan-Russia-Taiwan) joint MRSA studies started in 2006, and since then have isolated PVL^+^ ST30/*spa*19(t019)/SCC*mec*IVc CA-MRSA (strain RS08), only one precisely confirmed PVL^+^ case in Russia, from a 23-year-old female badminton player with furunculosis in Vladivostok, Far Eastern Russia [40]. We analyzed the whole genome structures, in terms of comparative genomics, of two unique Russian ST239/SCC*mec*III lineages: *spa*351(t030)/SCC*mec*III_R_ (strain 16K) from a case of urethritis in Vladivostok [41] and *spa*3(t037)/SCC*mec*IIIA (ST239_Kras_ strain OC3) from a case of fatal pneumonia with sepsis in Krasnoyarsk, Siberian Russia [42]. The latter, ST239_Kras_, represented the Siberian Russian clade [42] of the globally important ST239 HA-MRSA lineage [39].

In the present study, we determined the complete circular genome sequence of prevalent ST8/SCC*mec*IVc CA-MRSA (ST8_Kras_ strain OC8 [42]), which was isolated from a fatal pediatric pneumonia case in Krasnoyarsk, Siberian Russia. Based on OC8 data, we found that ST8/SCC*mec*IVc MRSA, which has widely spread in Russia, including European, Siberian, and Far Eastern regions, commonly carried a characteristic large (one-megabase) genomic inversion (MbIN), triggered by IS*256* at hot spots, thereby establishing a novel unique clade (Russia ST8-IVc) of the global ST8/SCC*mec*IV CA-MRSA lineage. The evolution, potential virulence, and selective advantages of Russia ST8-IVc and also IS*256*’s spread and functions were discussed.

## Materials and Methods

### Ethics statement

The Ethics Review Boards of Krasnoyarsk State Medical University (Ethics Review Board No28/2010), Krasnoyarsk, Russia; Far Eastern Federal University School of Biomedicine, Vladivostok, Russia, together with the International Medical Education and Research Center, Niigata, Japan (Ethics Review Board No66-01-17/152) and the National Taiwan University College of Medicine, Taipei, Taiwan, specifically approved this study. Written informed consent was obtained from patients, where necessary.

### Bacterial strains

Twenty-five MRSA strains were used in this study and data, including those described previously [11,41–43], are summarized in Table 1. The epidemiological definitions of CA-MRSA and HA-MRSA were based on the Centers for Disease Control and Prevention (CDC) criteria [2]. MRSA from Siberian Russia (Krasnoyarsk) included 10 strains of ST8/SCC*mec*IVc CA-MRSA (ST8_Kras_) from cases of SSTIs, community- or hospital-acquired pneumonia (CAP or HAP), sepsis, colitis, and healthy carriers (students and hospital workers) [42]; of these, strain OC8, which was isolated from a case of fatal pediatric CAP, was subjected to a complete genome sequence analysis in the present study. Strain OC3 of ST239/SCC*mec*IIIA HA-MRSA (ST239_Kras_), which was isolated from a case of fatal adult HAP with sepsis, and the comparative genome of which was analyzed [42], was also employed. MRSA from European Russia (Moscow, St. Petersburg, and Yaroslavl) were eight ST8/SCC*mec*IVc strains from cases of SSTIs, sepsis, osteomyelitis, fatal HAP, and a healthy carrier (hospital worker). MRSA from Far Eastern Russia (Vladivostok) included three strains of ST8/SCC*mec*IVc CA-MRSA isolated from cases of urethritis and SSTIs [41], and an additional ST8/SCC*mec*IVc strain from a case of nosocomial respiratory tract infection. ST239/SCC*mec*III_R_ strain 16K, which was isolated from a case of urethritis and the comparative genome of which was analyzed [41], was also employed. ST30/*spa*19(t019)/SCC*mec*IVc strain RS08, which was isolated in 2006 in Vladivostok [40], was used as a reference strain of Russian CA-MRSA.

**Table 1:** Relevant characteristics of MRSA strains^a^.

Isolation		Strain	Isolation year	Genotype:	Gene for toxin^a^, superantigen, adhesin^a^	Resistance^a^^,^^b^			Patient				Reference
country,				ST, *spa*^a^, *agr*,		β-lactam agent		Non β-lactam agent^a^	Disease^a^	Outcome^a^	Age^a^	Sex^a^	
region,				SCC*mec*^a^, Coa		MIC (μg/ml)							
city^a^						OXA^a^	IPM^a^						
Russia													
Siberian	K^a^	OC1	2010	8, 1 (t008), 1, IVc^a^, III	*sea*, *psmα*(↑)^a^	32	0.13	G, K, E Cl, L, Ch, Su	Skin abscess	R	50Y	F	40
		OC1C	2011	8, 1 (t008), 1, IVc^a^, III	*sea*, *psmα*(↑)^a^	32	0.25	L, Ch	Colitis	R	3Y	M	40
		OC8	2007	8, 1 (t008), 1, IVc^a^, III	*sea*, *psmα*(↑)^a^	32	0.25	L, Ch	Pneumonia (CAP)	D	1Y	M	40
		OC11	2007	8, 1 (t008), 1, IVc^a^, III	*sea*, *psmα*(↑)^a^	32	0.25	L, Ch	Pneumonia, sepsis	D	39Y	M	40
		OC22	2008	8, 1 (t008), 1, IVc^a^, III	*sea*, *psmα*(↑)^a^	32	0.5	L, Ch	Pneumonia (CAP)	D	41Y	M	40
		OC23	2008	8, 1 (t008), 1, IVc^a^, III	*sea*, *psmα*(↑)^a^	64	0.13	L, Ch	Pneumonia (CAP)	D	40Y	M	40
		OC52	2008	8, 1 (t008), 1, IVc^a^, III	*sea*, *psmα*(↑)^a^	32	0.25	L, Ch	−^c^	−^c^	34Y	F	40
		OC59	2008	8, 1 (t008), 1, IVc^a^, III	*sea*, *psmα*(↑)^a^	32	0.13	G, K, L, Ch	Pneumonia	D	4M	F	40
		OC160	2011	8, 1 (t008), 1, IVc^a^, III	*sea*, *psmα*(↑)^a^	32	0.5	G, K, E, Cl^ind^, L, Ch	Wound infection, cellulitis	R	53Y	M	40
		OC217	2010	8, 1 (t008), 1, IVc^a^, III	*sea*, *psmα* (↑)^a^	32	0.13	L	−^d^	−^d^	19Y	F	40
		OC3	2007	239, 3 (t037), 1, IIIA^a^, IV	*ts*t, *sek*, *seq*,	≥256	64	G, K, E, Cl, L, T, Ch,	Pneumonia (HAP), sepsis	D	46Y	M	40
					*psmα*(↑)^a^, *cna*			R, Su, St					
European	M^a^	M79	2011	8, 1 (t008), 1, IVc^a^, III	*sea*, *psmα*(↑)^a^	32	0.25	G, K, L	Wound infection	R	20-60Y^e^	−^e^	This study
		M185	2008	8, 1580 (t2648), 1, IVc^a^, III	*sea*, *psmα*(↑)^a^	32	0.25	G, K, E, Cl, L, Ch	Sepsis	D	20-60Y^e^	−^e^	This study
		M257	2012	8, New (t1259), 1, IVc^a^, III	*sea*, *psmα*(↑)^a^	64	0.25	E, Cl, L, Ch	−^c^	−^c^	20-60Y^e^	−^e^	This study
	S^a^	S2	2011	8, 1 (t008), 1, IVc^a^, III	*sea*, *psmα*(↑)^a^	64	0.25	G, K, L, Ch	Burn wound	R	20-60Y^e^	−^e^	This study
		S14	2011	8, 363 (t024), 1, IVc^a^, III	*sea*, *psmα*(↑)^a^	64	1	G, K, E, Cl, L, Ch	Burn, sepsis	R	20-60Y^e^	−^e^	This study
		S65	2011	8, 1 (t008), 1, IVc^a^, III	*sea*, *psmα*(↑)^a^	64	0.25	L, Ch	Wound, osteomyelitis	R	20-60Y^e^	−^e^	This study
		S214	2012	8, 363 (t024), 1, IVc^a^, III	*sea*, *psmα*(↑)^a^	64	4	G, K, L, Ch	Osteomyelitis, pneumonia (HAP)	D	20-60Y^e^	−^e^	This study
	Y^a^	Y269	2011	8, 1 (t008), 1, IVc^a^, III	*sea*, *psmα*(↑)^a^	≥256	16	G, K, E, Cl, L, Ch	Wound infection	R	20-60Y^e^	M	This study
Far Eastern	V^a^	12K	2008	8, 826 (tUK)^a^, 1, IVc^a^, III	*sea*, *psmα*(↑)^a^	32	0.06	G, K, Ch	Urethritis	R	21Y	M	41, this study
		40K	2008	8, 826 (tUK)^a^, 1, IVc^a^, III	*sea*, *psmα*(↑)^a^	32	0.06	G, K, E, Cl, Ch	Wound infection	R	19Y	M	41, this study
		RF57^f^	2006	8, 826 (tUK)^a^, 1, IVc^a^, III	*sea*, *psmα*(↑)^a^	64	1	G, K, E, Cl, Ch, R	Wound infection	R	20-60Y^e^	−^e^	41, this study
		RF570	2010	8, 826 (tUK)^a^, 1, IVc^a^, III	*sea*, *psmα*(↑)^a^	64	1	G, K, E, Cl, Ch, R	Nosocomial respiratory tract infection	R	20-60Y^e^	−^e^	This study
		16K	2008	239, 351 (t030), 1, III_R_^a^, IV	*sea*, *sek*, *seq*, *cna*	≥256	64	G, K, E, Cl, L, T, Ch, R, Su, St	Urethritis	R	20Y	M	41, this study
USA		USA300-0114	-^g^	8, 1 (t008), 1, IVa^a^, III	PVL^+^^a^, ACME^+^^a^, *sek*, *seq*, *psmα*(↑)^a^	32	0.13	K, E, Ci^i^, T	-^g^	-^g^	-^g^	-^g^	11, 42, 43

^a^City: K, Krasnoyarsk; M, Moscow; S, St. Petersburg; Y, Yaroslavl; V, Vladivostok. SCC*mec*: IVc, IV.3.1.2; IVa, IV.1.1.1; IIIA, III.1.1.2; III_R_, III.1.1.4. *spa* type: tUK (Unknown), unknown Ridom *spa* number. Gene: *psmα*(↑), elevated *psmα* expression (which was significantly higher than HA-MRSA expression levels, *P*<0.05). Other common adhesin genes include *lukE-lukD*, *hla*, *hlg*, *hlg-v*, *hld*, split *hlb*, and *c12ag* (*icaA*, *icaD*, *eno*, *fnbA*, *fnbB*, *ebpS*, *clfA*, *clfB*, *fib*, *sdrC*, *sdrD*, *sdrE*). PVL, Panton-Valentine leukocidin; ACME, arginine catabolic mobile element. Antimicrobial agent: OXA, oxacillin; IPM, imipenem; G, gentamicin; K, kanamycin; E, erythromycin; Cl, clindamycin; L, levofloxacin; Ci, ciprofloxacin; T, tetracycline; Ch, chloramphenicol; R, rifampicin; Su, sulfamethoxazole; St, streptomycin; Resistance: ind, inducible resistance; i, intermediate. Pneumonia: CAP, community-acquired pneumonia; HAP, hospital-acquired pneumonia. Outcome: R, recovery; D, death. Age: Y, year; M, month. Sex: F, female; M, male.

^b^Levofloxacin-resistant strains were also resistant to ciprofloxacin.

^c^Healthy carrier case (hospital worker).

^d^Healthy carrier case (student).

^e^Full personal (patient) information, not available.

^f^Previous name, 57H.

^g^No description.

USA300-0114, a type strain of ST8/SCC*mec*IVa CA-MRSA (USA300) from USA [11], was kindly provided by L. K. McDougal and L. L. McDonald.

### Genotyping and virulence gene analysis

The molecular typing of MRSA, such as ST, clonal complex (CC), *spa*, *agr*, SCC*mec* [13], and Coagulase (Coa), was performed as described previously [42,44]. Regarding *spa*, allele numbers and types were determined using the public *spa* type databases, eGenomics (http://tools.egenomics.com/) and Ridom SpaServer (http://spaserver.ridom.de/). Forty-nine virulence genes were analyzed by PCR [44]: 3 leukocidin genes (*luk*_*PV*_*SF*, *lukE-lukD*, and *lukM*), 5 hemolysin genes (*hla*, *hlb*, *hlg*, *hlg-v*, and *hld*), the peptide cytolysin, PSMα (*psmα*), 19 staphylococcal superantigen (SAg) genes, named enterotoxin (SE) or enterotoxin-like (SEl) (*tst*, *sea*-*e*, *seg*-*j*, *selk*-*r*, and *selu*), staphylococcal exotoxin (*set*) genes, a staphylococcal superantigen-like gene cluster (*ssl*), 3 exfoliative toxin genes (*eta*/*b* and *etd*), the epidermal cell differentiation inhibitor gene (*edin*), 14 adhesin genes (*icaA*/*D*, *eno*, *fib*, *fnbA*/*B*, *ebpS*, *clfA*/*B*, *sdrC*-*E*, *cna*, and *bbp*), and the ACME-*arcA* gene.

### Pulsed-field gel electrophoresis (PFGE) analysis

Bacterial DNA for PFGE was digested with *Sma*I and electrophoresed in 1.2% agarose with marker DNA (Lambda ladder; Bio-Rad Laboratories, Inc., Hercules, CA, USA), as described previously [41,42].

### Susceptibility testing

Susceptibility testing of bacterial strains was performed using the agar dilution method with Mueller-Hinton agar [45]. Inducible clindamycin resistance was tested, as above, by using agar plates containing erythromycin at 1 μg/ml [42].

### Genome analysis

The OC8 genome was analyzed by a long-read single-molecule real-time (SMRT) sequencing platform with P5/C3 chemistry using sequencing technology, a PacBio RS II system (Pacific Biosciences, Menlo Park, CA, USA), with the assembler software SMRT Analysis v2.3.0/hierarchical genome-assembly process (HGAP) pipeline [46]. Genome coverage (sequencing depth) was 259-fold of the genome size. Finishing of the genome contig to construct the complete circular genome sequence was performed by PCR and sequencing. The GenBank accession number for the OC8 complete circular genome sequence is AP017377.

### Pairwise comparison between two genome sequences

In the inversion analysis, pairwise comparisons between two MRSA genome sequences were performed using WebACT (http://www.webact.org/WebACT/home).

### Homology analysis

A homology analysis was performed using software BLAST (http://blast.ddbj.nig.ac.jp/top-e.html).

### mRNA expression assay

The mRNA expression levels of the *psmα* gene and 16S rRNA genes were examined using an RT-PCR assay [42,47]. *psmα* expression levels were normalized to 16S rRNA expression levels. ST5/SCC*mec*II HA-MRSA strains (N315 and Mu50) were used as low *psmα* expression control strains, and the ST8/SCC*mec*IVa CA-MRSA type strain USA300-0114 and ST30/SCC*mec*IVc CA-MRSA strain RS08 were used as elevated *psmα* expression control strains [42].

### Statistical analysis

Data were evaluated by Fisher’s exact test and an analysis of variance with repeated measurements for the mRNA expression assay. The level of significance was defined as a *P* value of <0.05.

## Results

### Molecular characteristics of ST8 MRSA in Russia

**Fig 1:**
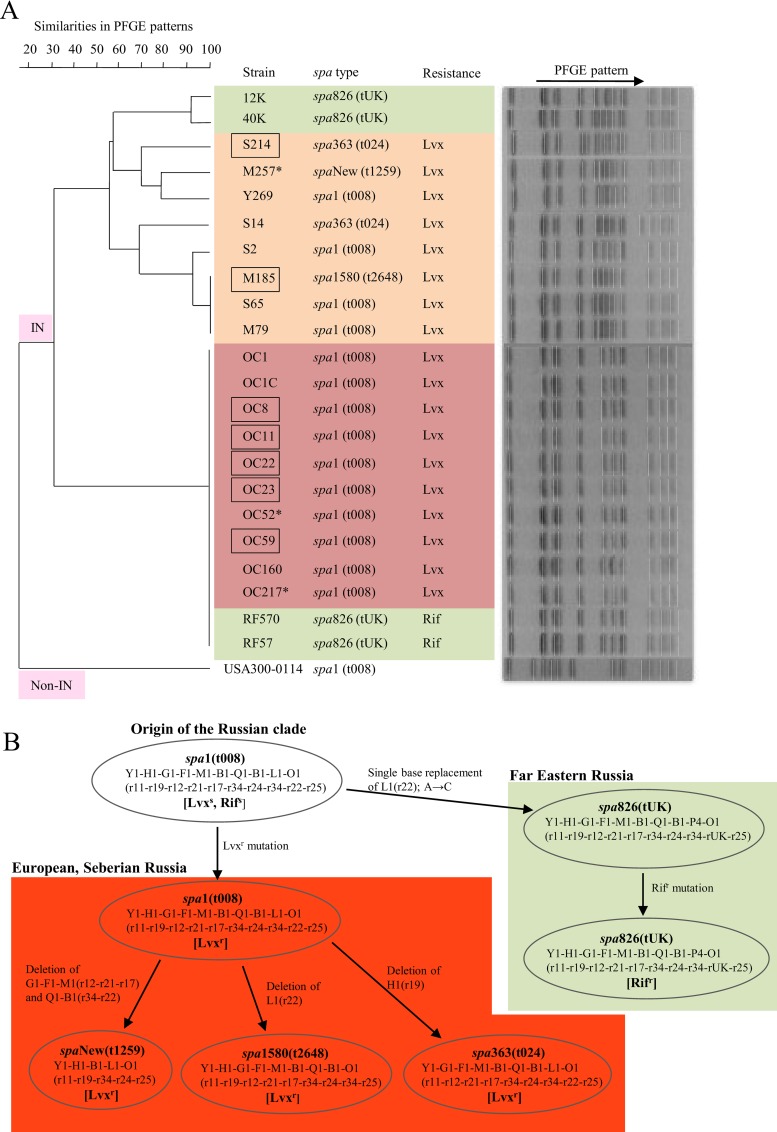
**Pulsed-field gel electrophoresis (PFGE) analysis (A) and phylogenetic *spa* type analysis of (B) of ST8/SCC*mec*IVc MRSA strains isolated in Russia.** The MRSA strains shown are those described in Table 1. In A, strains were classified into two major groups: IN, those with a megabase inversion, and non-IN, those without a megabase inversion. The geographical location of MRSA isolation is colored: yellow, European Russia (Moscow, St. Petersburg, and Yaroslavl); red, Siberian Russia (Krasnoyarsk); green, Far Eastern Russia (Vladivostok). Square, isolated from a fatal case; asterisk, isolated from a healthy carrier. tUK, tUnknown (unknown Ridom *spa* number). Lvx, levofloxacin; Rif, rifampicin. In B, *spa* allele numbers and Ridom *spa* repeat numbers (in parentheses) are both shown. *spa*1(t008) represents the ancestral *spa* type for Russian ST8/SCC*mec*IVc MRSA; other *spa* types diverged directly from the common ancestral type. Lvx^s^, levofloxacin-susceptible; Rif^s^, rifampicin-susceptible; Lvx^r^, levofloxacin-resistant; Rif^r^, rifampicin-resistant.

The molecular characteristics of ST8 MRSA strains from European Russia (Moscow, St. Petersburg, and Yaroslavl), Siberian Russia (Krasnoyarsk), and Far Eastern Russia (Valdivostok) are summarized in Table 1 and Fig 1. All ST8 strains exhibited the same genotypes for *agr*1, SCC*mec*IVc, and CoaIII. *spa* types were divergent depending on geographical locations. *spa*1(t008) was likely the common type, accounting for 100% (10/10) for Siberian Russia and 50% (4/8) for European Russia, but 0% (0/4) for Far Eastern Russia. Based on this result, together with previous findings showing that *spa*1(t008) was the most prevalent type [38], *spa*1(t008) may be the ancestral ST8 *spa* type (Fig 1B). All ST8 strains were positive for *sea*.

All ST8 strains exhibited elevated *psmα* expression, similar to CA-MRSA (USA300 and RS08). Their MIC values for oxacillin and imipenem were lower than those of HA-MRSA (for example, OC3 and 16K), which is consistent with the characteristics of CA-MRSA [36], however, the MIC of imipenem for the Yaroslavl case was high (16 μg/ml). Therefore, the ST8 MRSA strains met the bacteriological criteria for CA-MRSA, although strains OC11, OC52, M257, S214, RF57, and RF570 were isolated from inpatients or hospital workers (healthy carriers), suggesting their spread even in hospitals.

All ST8 strains from Siberian and European Russia were resistant to levofloxacin (MICs, 4–8 μg/ml), while ST8 strains from Far Eastern Russia were susceptible. Only one strain from Siberian Russia (OC160) exhibited inducible clindamycin resistance; other cases of clindamycin resistance were constitutive. Rifampicin resistance (MICs, 4 μg/ml) was only detected in Far Eastern Russia. Chloramphenicol resistance (MICs, 64 μg/ml) was a common feature. Regarding diseases, of the ST8 strains analyzed, seven were from fatal cases of pneumonia or sepsis (Table 1).

In the PFGE analysis (Fig 1A), ST8 strains from Siberian Russia constructed a single cluster, suggesting the spread of a single type (ST8_Kras_). ST8 strains from European Russia were divergent from ST8_Kras_, and constituted some heterogeneous clusters. ST8 strains from Vladivostok included the ST8_Kras_ type and a divergent type, which clustered within the European Russia types.

### The circular genome structure of ST8_Kras_ strain OC8

**Fig 2:**
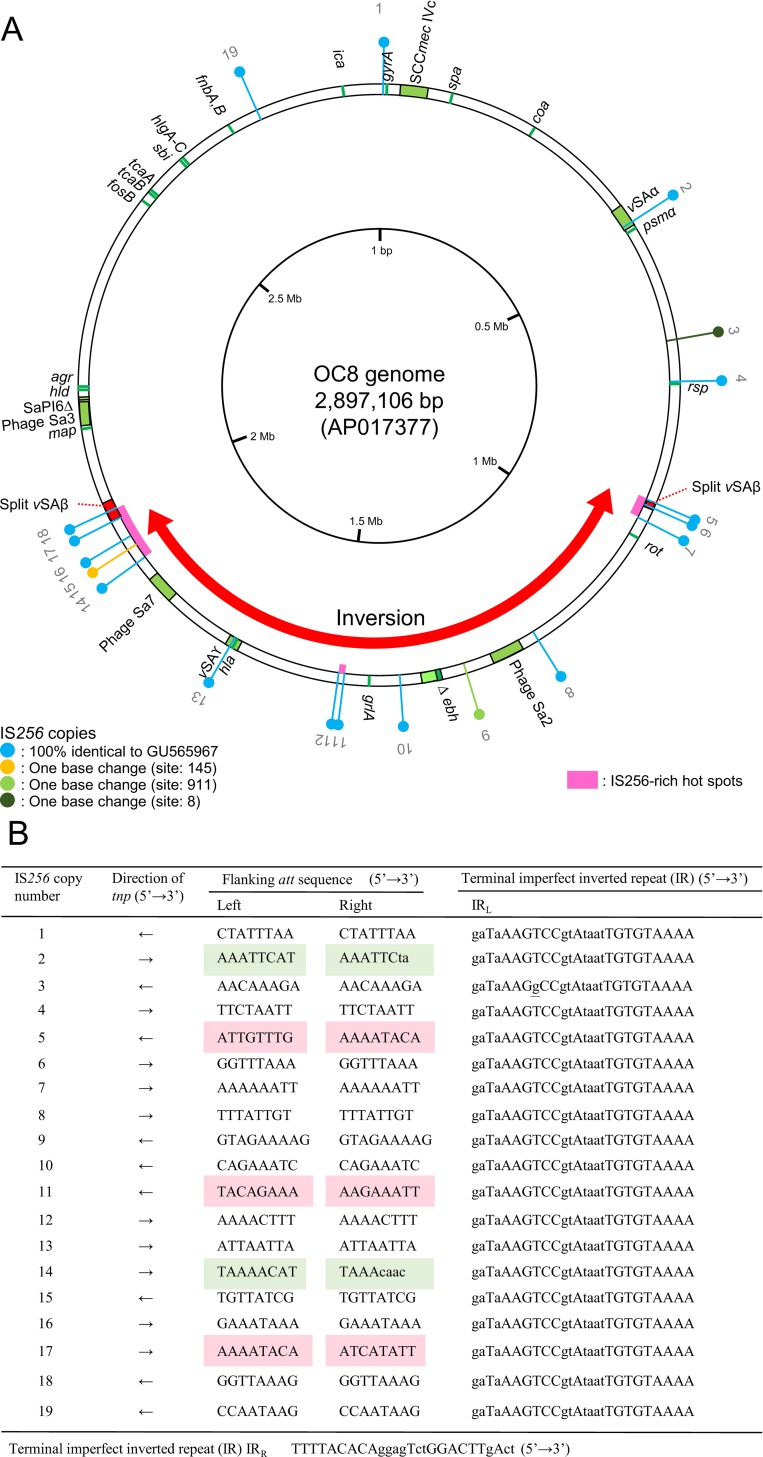
**OC8 circular genome map (A) and genetic status of IS*256* copies on the genome (B).** In A, OC8 genome information includes MRSA-typing targets, phages, SaPIs, mobile genetic elements, including IS*256*, virulence, drug resistance, and inversion. Genes (products) described on the genome map are: *spa*, protein A; *coa*, coagulase; *psmα*, phenol-soluble modulin *α* (cytolytic peptide); *rsp*, AraC family transcriptional regulator; *rot*, repressor of toxins; *ebh*, extracellular matrix-binding protein (very large surface-anchored protein/giant protein); *grl*, DNA topoisomerase IV (quinoplone resistance); *hla*, α-hemolysin (Hla); *map*, map protein; *hld*, δ-hemolysin (Hld); *agr*, accessory gene regulator; *fos*, fosfomycin resistance protein; *tca*, teicoplanin resistance-associated membrane protein; *sbi*, IgG-binding protein; *hlg*, γ-hemolysin (Hlg); *fnb*, fibronectin-binding protein; *ica*, intercellular adhesion protein A (biofilm formation); *gyr*, DNA gyrase (quinoplone resistance). The staphylococcal complement inhibitor (SCIN) gene (*scn*), staphylokinase (SAK) gene (*sak*), and superantigen SEA gene (*sea*) were carried by phage Sa3, and the β-hemolysin (Hlb) gene (*hlb*) was split by a phage Sa3 insertion. The OC8 genome carried 19 copies of IS*256*; they are numbered (① to ⑲), as shown in the figure. IS*256*-enriched hot spots are marked in pink. A large genomic inversion (MbIN), relative to USA300 FPR3757 (GenBank accession number CP000255), occurred between IS*256*⑤ and IS*256*⑰; the inverted region is marked with a red thick arrow. Due to MbIN, the genomic island vSAβ, which carried three IS*256* (⑥, ⑰, and ⑱), was split into two parts located far from each other. In B, the direction of the IS*256* insertion is shown by arrows. Attachment (*att*) site sequences appear on both sides of IS*256* as direct repeats (DRs) upon insertion [48,49]; the *att* sequences of 19 IS*256* copies were all divergent from each other. The *att* sequences in capital letters were present as *att* at the corresponding position of USA300 FPR3757, which lacked IS*256*. Regarding unusual *att* sets, the red mark (box) indicates heterogeneous *att* sequences on the left and right sides, and the green mark indicates the imperfect DRs of *att*. The 26-bp imperfect terminal inverted repeats of IS*256* were identical for 19 IS*256* copies, except for IS*256*③, which had one base change.

The OC8 genome was estimated to be 2,897,106 bp, sharing an approximately 99.9% homologous core region with the USA300 FPR3757 genome (GenBank Accession Number CP000255), albeit with highly diverged regions, such as phages and mobile genetic elements. Moreover, strain OC8 carried a 2,908-bp chloramphenicol resistance plasmid (pOC8) [42]. Based on the OC8 complete circular genome sequence, the OC8 circular genome map was constructed, as shown in Fig 2A, with a focus on phages, SaPIs, genomic islands, insertion sequences (particularly IS*256* [48,49]), resistance genes or mutations, some virulence genes, some regulatory genes or regulons, and genes and genetic structures used for genotyping (*spa*, *agr*, *coa*, SCC*mec*). OC8 lacked drug resistance transposons; for example OC8 lacked Tn*4001* and Tn*554*, in marked contrast to the ST237 HA-MRSA lineage in Russia (strains OC3 [42] and 16K [41]).

Regarding phages, the OC8 genome carried φSa2. φSa2 (OC8) was 45,781 bp in size and showed 86.5% homology to PVL-converting φSa2 (USA300 FPR3757), but lacked the PVL genes. The second phage was φSa7 of 44,446 bp in size. φSa7 (OC8) had no virulence genes. USA300 FPR3757 lacked φSa7. The third phage was φSa3 of 42,984 bp in size. φSa3 (OC8) was inserted into the *hlb* gene (Fig 2A). As shown in S1 Fig, φSa3 (OC8) had the immune evasion cluster (IEC) with the immune evasion genes *scn* (for staphylococcal complement inhibitor, SCIN) and *sak* (for staphylokinase, SAK) and also the SAg gene *sea*, on the left-end side, similar to ST239/SCC*mec*III HA-MRSA TW20, which was isolated from a case of intensive care unit (ICU)-associated bacteremia in London [50,51]. Although the overall homology between φSa3 (OC8) and φSa3 (TW20) was 89.8%, similarities with the *scn*, *sak*, and *sea* genes were high at 99.4%, 99.8%, and 100%, respectively. The IEC of φSa3 (USA300 FPR3757) carried *sak*, *chp* (for the chemotaxis inhibitory protein of *S*. *aureus*, CHIPS), and *scn*, but lacked *sea*. φSa3 (OC8) and φSa3 (USA300 FPR3757) showed a homology of 81.8%.

Regarding SaPI, the OC8 genome carried SaPI6∆ with no SAg gene, and lacked SaPI5 carrying *sek* and *seq*, which was present in USA300 [10]. The OC8 genome lacked SaPI-carrying SAg genes.

**Fig 3:**
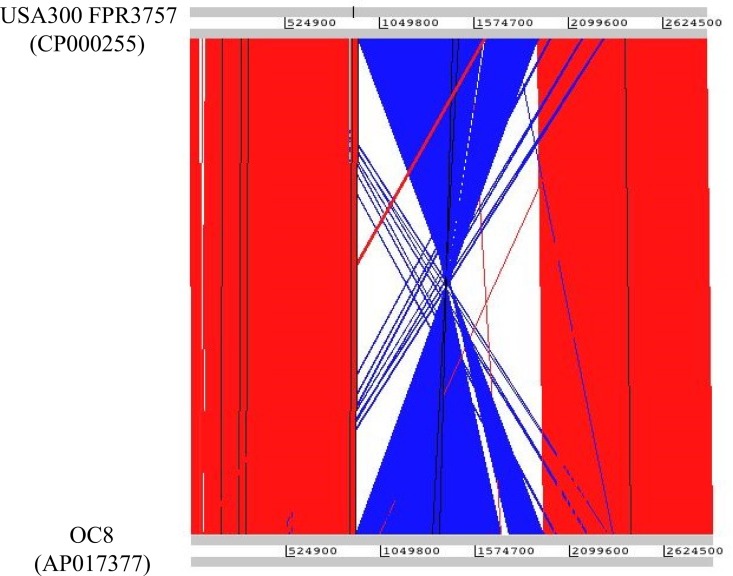
Sequence comparison between OC8 and USA300 FPR3757 genomes and visualization of a large genomic inversion. Genomic sequence comparisons were performed using WebACT for the visualization of genomic inversions. The genome sequence of USA300 FPR3757 was from GenBank Accession number CP000255. The OC8 inverted region relative to USA300 FPR3757 is highlighted in blue.

Regarding insertion sequences, 19 copies of IS*256* were distributed along the OC8 genome. Their distribution was not random; there were three IS*256*-enriched regions, reflecting the gathered regions of IS*256*-preferred insertion sites (Fig 2A). These IS*256*-enriched regions may serve as recombination hot spots. This was in marked contrast to the USA300 FPR3757 genome, which did not have IS*256* [30]. A large genomic inversion was identified relative to the USA300 FPR3757 genome; this event was triggered by two IS*256* copies (⑤ and ⑰) in the hot spots, as shown in Fig 2A and visualized in Fig 3. The large genomic inversion was 1,042,885 bp in size, and corresponded to 36.0% of the OC8 genome; this approximately one-megabase genomic inversion was abbreviated as MbIN.

In addition to IS*256*, the OC8 genome carried some other insertion sequences: IS*431mec* and ∆IS*1272* in SCC*mec*IVc; two copies of IS*1181* (of those, one copy had ∆*tnp*); and tree copies of IS*200* family, which showed a 90.2% homology to ISSep3-like, therefore, suggesting that IS*256* was the most prevalent insertion sequence on the OC8 genome. The OC8 genome did not have a *ccrC*-carrying unit, which was found in the ST59/SCC*mec*V(5C2&5) CA-MRSA from Taiwan [21] and also distributed to the ST239/SCC*mec*III_R_ HA-MRSA lineage from Russia [41].

Regarding genomic islands, the OC8 genome carried 33,301-bp vSAα with an IS*256*② insertion. The second genomic island was vSAβ, which was 35,235 bp in size and showed 99.8% homology to vSAβ (USA300 FPR3757). vSAβ (OC8) contained three IS*256* insertions (IS*256*⑥, IS*256*⑰, and IS*256*⑱). Moreover, vSAβ (OC8) was split into two parts by a MbIN event between IS*256*⑤ and IS*256*⑰ (Fig 2A). The third genomic island was a 21,319-bp vSAγ with an IS*256*⑬ insertion.

Toxin genes on the OC8 genome map included *psmα*, *hla* (in vSAγ), *sea* (in φSa3), *hld*, and *hlg*. *hlb* was split due to a φSa3 insertion. Immune evasion genes, included in the map, were *spa*, *ebh*, *map*, *scn* and *sak* (in φSa3), *sbi*, and *fnbA*, *B*. Of those, *ebh* (encoding for the giant protein Ebh [52,53]) had a nonsense mutation (G→T at position 11,029 bp) and was shortened due to a prenature stop codon (TAA); thus, the truncated product of ∆*ebh* was predicted to be only 3,676 aa long, corresponding to 35.3% of the entire *ebh* gene product (Ebh, 10,421 aa long [53]) of USA300 FPR3757 (S2 Fig); ∆*ebh* is marked by dark green in Fig 2A.

Regarding IS*256* insertions, which may affect gene expression and regulation, IS*256*④ was inserted 521 bp upstream of *rsp* (a gene for the AraC family transcriptional regulator). There were no IS*256* insertions in *ica* (biofilm-associated gene cluster, *ica* operon), *rot* (gene for the repressor of toxins), or their promotor regions; IS*256* insertions in these genes (or promotor regions) were noted in terms of IS*256*-directed virulence alternations [30,34]. The location of *rot* on the OC8 genome was markedly divergent from *rot* (USA300 FPR3757) due to OC8 MbIN (Fig 2A).

Regarding drug resistance specified by the genome, the levofloxacin resistance of OC8 was due to *gyrA* (Ser84Leu) and *grlA* (Ser80Phe) mutations. OC8 carried the fosfomycin-inactivating enzyme gene (*fosB* [54]); however, the MIC of fosfomycin for OC8 was 1.0 μg/ml. There were no IS*256* insertions in *tcaA*,*B* (glycopeptide resistance-related genes), the inactivation of which resulted in glycopeptide resistance phenotypes [55,56].

### Status of multiple IS*256* copies in OC8

IS*256* has a 26-bp imperfect terminal IR, and is flanked by the direct repeat (DR) of 8 or 9-bp host *att* site sequences [27,48,49]. We analyzed the status of all 19 IS*256* copies on the OC8 genome; data are summarized in Fig 2A, B. All IS*256* copies shared the same or similar sequences; 16 out of the 19 copies were the same, and three had one nucleotide replacement (Fig 2A). Regarding 26-bp imperfect terminal IR sequences, only IS*256*③ had a single base change (Fig 2B).

The flanking *att* site sequences for 19 IS*256* copies in OC8 were 8 or 9 bp, and are summarized in Fig 2B. These flanking *att* site sequences were all divergent and generally AT-rich.

Regarding the arrangement of *att* sequences on the left and right (*att*L and *att*R), in 14 out of the 19 IS*256* copies (73.7%), *att*L and *att*R were the same (and directly oriented as DR), and such *att* sequences were present as an *att* site at the corresponding position of the USA300 FPR3757 genome, as expected. However, for two IS*256* copies (② and ⑭) (10.5%), *att*L and *att*R were imperfect repeats, and although the *att*L sequence was present as an *att* site at the corresponding position of USA300 FPR3757, no *att*R sequence was present in USA300 FPR3757. In the remaining three IS*256* copies (⑤, ⑪, and ⑰) (15.8%), *att*L and *att*R were heterogeneous; however, *att*L and *att*R were both present as *att* sites at the corresponding positions in USA300 FPR3757. The last three cases, IS*256*⑤, ⑪, and ⑰, were present in IS*256*-rich hot spots on the OC8 genome (Fig 2A).

Nine out of 19 IS*256* copies (47.4%) were inserted in the opposite orientation, as shown with, for example, IS*256*⑤ vs. IS*256*⑰ (Fig 2B). The OC8 genome had no Tn*4001*, which had two terminal IS*256* copies [33].

**Fig 4:**
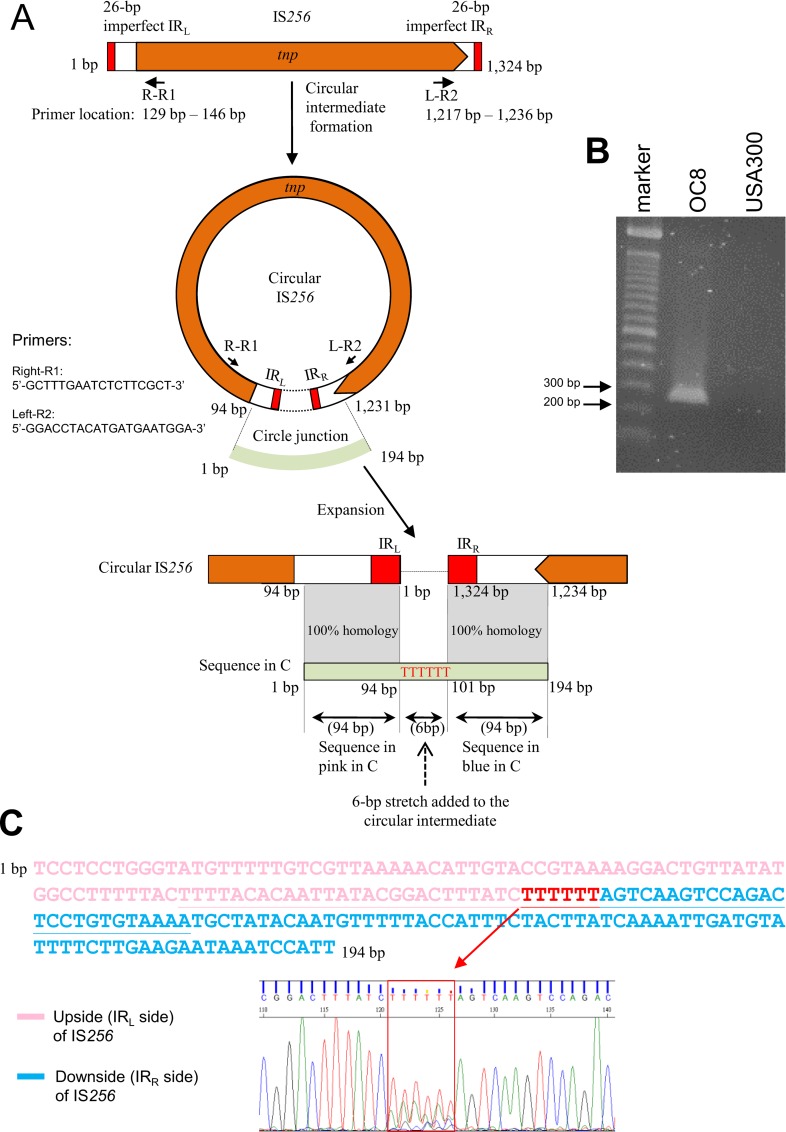
The structure of IS*256* and its extrachromosomal circular DNA in OC8. In A, the structure of IS*256* (OC8) is based on the OC8 genome sequence (GenBank accession number AP017377); the structure was very similar to previously described IS*256* structures [27,48,49]. PCR primers to detect an IS*256* circular DNA were designed based on the OC8 genome sequence. In B, the PCR primer set (R-R1 and L-R2, shown in A) exactly detected IS*256* circular DNA for OC8 (PCR product size, approximately 200 bp), while there were no amplified bands for strain USA300-0114, which lacked IS*256*. In C (and B), the 194-bp nucleotide sequence of the estimated PCR product, perfectly matched the IR_L_ side and IR_R_ side regions of IS*256* (OC8), and contained a 6-bp stretch, marked in red; 26-bp imperfect IR sequences and 6-bp stretch sequences were underlined in C. However, the 6-bp stretch data showed a “mixed” result, with TTTTTT as the highest base content (followed by AAAAAA). Since the 6-bp stretch originates from a flanking *att* sequence [48] and OC8 carries 19 IS*256* copies with distinct *att* sequences, the “mixed” 6-bp stretch reflects the presence of heterogeneous circular DNA (in terms of stretch sequences) in OC8. This observation is consistent with the AT-rich *att* sequences of 19 IS*256* copies on the genome.

The *S*. *aureus* heritage of IS*256* [27] includes an extrachromosomal IS*256* circular molecule [48,49]. This extrachromosomal circular DNA of IS*256* was present in OC8 (Fig 4A and 4B). The circle junction of the IS*256* circular DNA in OC8 contained complete IS*256* termini, including imperfect IR_L_ and IR_R_, and an additional 6-bp nucleotide stretch. However, the 6-bp sequence determined was a mixture of distinct stretches, suggesting the presence of heterogeneous IS*256* circular molecules in OC8, each with a 6-bp stretch of a distinct sequence (Fig 4C).

### Genomic inversion and deletion triggered by IS*256*

**Fig 5:**
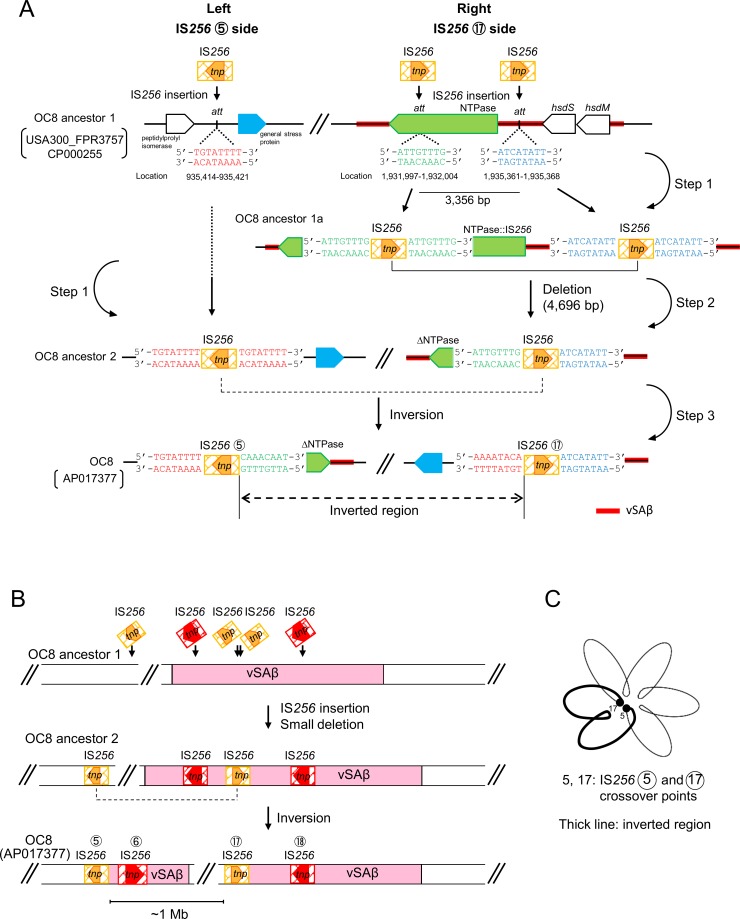
Possible mechanisms for the large genomic inversion in OC8. In this model, shown in A, we hypothesized ancestor strains of OC8 for a one-megabase inversion (MbIN) and simultaneously-occurring deletion events. An initial ancestor strain (OC8 ancestor 1) lacks IS*256*, but has *att* site sequences, similar to USA300 FPR3757 (GenBank accession number CP000255); the size of OC8 ancestor 1 DNA flanked by two *att* sites on the right side of the figure was estimated to be 3,356 bp. The first step (step 1) includes three IS*256* insertions at different *att* sites. As shown on the right side of the figure, a homogenous recombination (step 2) then occurs between the direct repeats of IS*256* (in OC8 ancestor 1a), deleting a small region and leaving only one copy of IS*256* (generating OC8 ancestor 2). In step 3, a homogenous recombination subsequently occurs between the inverted repeats of IS*256* (on OC8 ancestor 2), with the one-megabase region being inverted, and generating OC8. The genes of NTPase, *hsdS*, and *hsdM* (on the top right side) were located in the genomic island vSAβ (marked with a red line). In B, figures focus on a vSAβ split event, which occurred simultaneously with MbIN. OC8 ancestor 1, OC8 ancestor 2, and OC8 are the same as those described in A. In C, a hypothetical folded chromosome structure with loop domains is illustrated, based on [57], to boost the crossover and subsequent MbIN events at the two genomic locations, which are far from each other. (The diagram is not to scale.)

A possible model for the OC8 genomic inversion event triggered by two IS*256* (⑤ and ⑰) is shown in Fig 5. These events included two major steps: a deletion between the DR sequences of IS*256* and an inversion between the IR sequences of IS*256*. Furthermore, we hypothesized the presence of ancestor strains of OC8 (OC8 ancestor 1 and OC8 ancestor 2) in this model. On the left of Fig 5A, a single IS*256* insertion event occurred at the *att* site (5’-TGTATTTT) of OC8 ancestor 1, which was also present at the corresponding position of USA300 FPR3757, generating IS*256* flanked by DR of the *att* sequence (OC8 ancestor 2).

On the right of OC8 ancestor 1, two IS*256* insertion events possibly occurred at the two *att* sites (5’-ATTGTTTG and 5’-ATCATATT), which were also present at the corresponding positions of USA300 FPR3757, generating two IS*256* copies, which were flanked by each *att* DR (as shown in OC8 ancestor 1a). This may have been followed by homologous recombination between the two IS*256*, directly oriented (as DR), resulting in one IS*256* copy flanked by heterogeneous *att* sequences (5’-ATTGTTTG and 5’-ATCATATT), with a 4,696-bp deletion (3,356-bp OC8 ancestor 1 DNA plus 1,324-bp IS*256* DNA plus 16-bp left and right *att* sequences), as shown in OC8 ancestor 2.

Homologous recombination may have occurred between two IS*256*, oppositely oriented (as IR), in OC8 ancestor 2, resulting in current OC8 with two IS*256* copies (⑤ and ⑰), but with a 1,042,885-bp inside region inverted; this step essentially included the vSAβ split (Fig 5A and 5B). Fig 5C shows a hypothetically folded chromosome structure for OC8, potentially allowing for the crossover of two IS*256* copies (⑤ and ⑰), which are located approximately a distance of 1 Mb from each other; a figure was illustrated based on [57].

IS*256*⑪, with heterogeneous *att*L and *att*R and located at hot spots (Fig 2A and 2B), may have been the result of homologous recombination between two hypothetical IS*256* DR sequences in an OC8 ancestor strain, deleting a 1,403-bp region (63-bp OC8 ancestor 1 DNA plus 1,324-bp IS*256* DNA plus 16-bp left and right *att* sequences), as shown in S3 Fig.

### PCR detection and geographical distribution of MbIN

**Fig 6:**
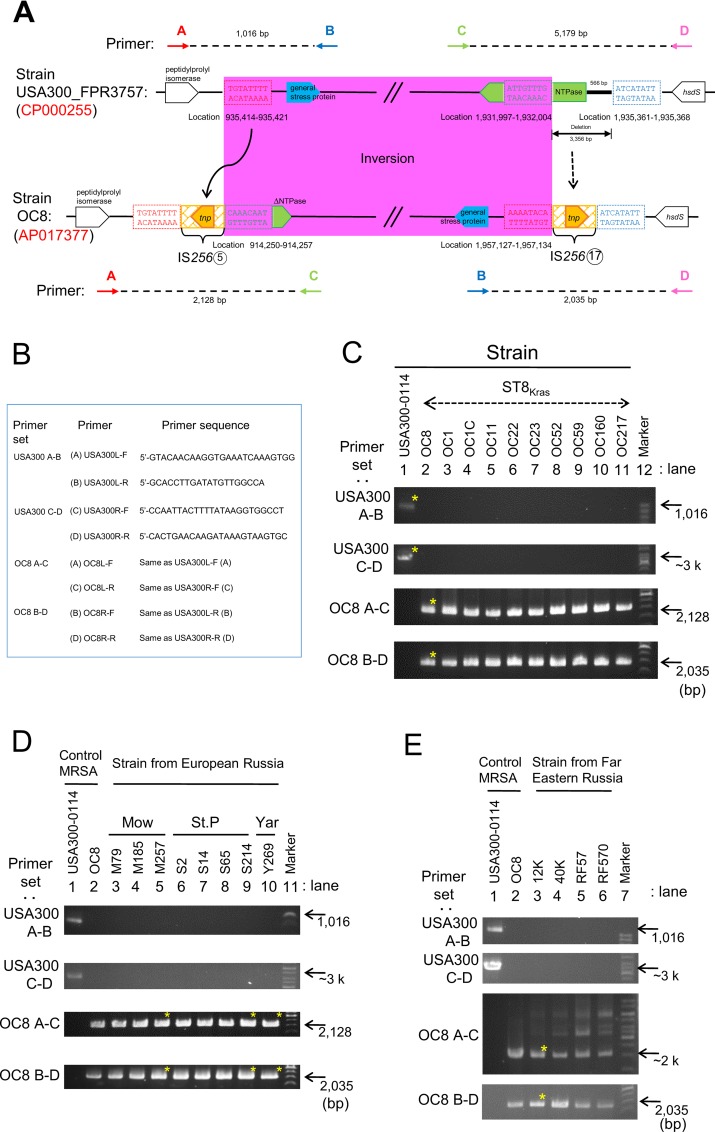
PCR targeting the OC8-type megabase inversion (MbIN). In A and B, PCR primers targeting the junction sites of OC8 MbIN (A-C and B-D) and those targeting the corresponding region of USA300 FPR3757 (A-B and C-D) were designed based on the OC8 or USA300 FPR3757 complete genome sequence, respectively. The structures of the MbIN junction regions of OC8 and the corresponding regions of USA300 FPR3757 are from Fig 5. In C to E, PCR products with an asterisk were sequenced, and the sequences determined were consistent with the OC8 or USA300 FPR3757 genome sequence. ST8_Kras_ is ST8/SCC*mec*IVc MRSA from Krasnoyarsk, Siberian Russia. The geographical location of MRSA isolated in European Russia: Mow, Moscow; St. P, St. Petersburg; Yar, Yaroslavl. Regarding Far Eastern Russia, MRSA was isolated in Vladivostok.

The OC8 genome has the characteristic junction regions of MbIN. In order to detect the left-side and right-side junction regions by PCR, PCR primers (A-C and B-D, respectively) were designed based on the OC8 complete genome sequence, as shown in Fig 6A and 6B. In order to detect the corresponding non-IN region of USA300 FPR3757, we designed PCR primers (A-B and C-D, respectively) based on the USA300 FPR3757 complete genome sequence (Fig 6A and 6B). PCR with the primer sets (A-B) and (C-D) gave positive bands for USA300-0114, as expected (Fig 6C), and the sequences of the PCR products were consistent with the USA300 FPR3757 sequences. The primer sets (A-B and C-D) produced negative results for OC8, as expected (Fig 6C). Instead, PCR with the primer sets (A-C) and (B-D) gave positive bands for the predicted sequences of OC8 and produced negative results for USA300-0114 (Fig 6C).

We then examined ST8/SCC*mec*IVc strains from European, Siberian, and Far Eastern Russia (Fig 6C to 6E). PCR with the primer sets (A-C) and (B-D) gave positive bands for the predicted sequences of all strains examined. PCR with the primer sets (A-B) and (C-D) produced negative results for all strains examined. These results strongly indicated that all ST8/SCC*mec*IVc strains from European, Siberian, and Far Eastern Russia carry MbIN, suggesting a common origin (with MbIN) in evolution.

### Analysis of the Russian ST239 HA-MRSA lineage for MbIN and IS*256* extrachromosomal circular DNA

Finally, the ST239 HA-MRSA lineage in Russia was examined for MbIN and IS*256* circular DNA by PCR. Regarding MbIN, when ST239/*spa*3(t037)/SCC*mec*IIIA HA-MRSA (strain OC3 from Krasnoyarsk) and ST239/*spa*351(t030)/SCC*mec*III_R_ HA-MRSA (strain 16K from Vladivostok) were examined, both strains were negative in PCR (A-C) and (B-D), but were positive in PCR (A-B) and (C-D), except for OC3, which was negative in PCR (C-D), most probably due to a mutation(s). Therefore, the Russian ST239 HA-MRSA lineage carried no OC8-type MbIN.

Regarding IS*256* circular DNA, the circle junction of IS*256* circular DNA in OC3 and 16K contained the complete IS*256* termini, including imperfect IR_L_ and IR_R_, and an additional 6-bp heterogeneous stretch (S4 Fig), similar to an OC8 case (albeit with distinct mixed sequence patterns due to a 6-bp heterogeneous stretch).

## Discussion

Inversions occur through homologous recombination, in which two genetic structures with homologous sequences of 300 bp or more are present in opposite orientations (as IRs) [58,59]. These genomic inversions (intrachromosomal recombination) are events involved in evolution; the genes in the inverted segment are functional [60] and inversions may create a selective advantage for bacterial pathogenesis, as reported with *Pseudomonas aeruginose* [61]. Large genomic inversions have been reported in ribosomal RNA genes (*rrn*) in *Escherichia coli* [62], in prophage regions in enterohemorrhagic *E*. *coli* serotype O157:H7 [63], in IS*6100* in *P*. *aeruginose* [61], in *Salmonella* Typhimurium [60], and also in *S*. *aureus* (MRSA USA800) [64]. *S*. *aureus* generally maintain the overall gene orders of the genome; however, in USA800 (ST5/SCC*mec*IV lineage), the genomic inversion relative to USA300 is approximately 500 kb in size, and may have occurred between IRs of IS*1181* and a 73-bp sequence [64].

The inversion in the present study (OC8) was triggered by IRs of IS*256* and was 1,042,885 bp in size, which was approximately two-fold larger than the USA800 genomic inversion, representing the largest genomic inversion in *S*. *aureus* (or MRSA). MbIN serves as an epidemiological marker in PCR targeting Russia ST8-IVc. The results of the present study also suggest that in addition to IRs of long homologous sequences, an additional factor, namely, IS*256*-enriched hot spots, is necessary for a large genomic inversion (MbIN) because even though there were several sets of IS*256* IRs on the OC8 genome, MbIN only occurred between those in IS*256*-enriched hot spots.

Deletion events occur when two long homologous sequences are directly oriented (as DRs) [59]. We found the trace of two deletions for OC8 of 63 bp and 3,356 bp in size, compared to the USA300 FPR3757 genome. These relatively small deletions also occurred at hot spots, at which IS*256* DRs were adjacent to each other. Therefore, an ancestral strain of OC8 (before deletion events) may have carried two more IS*256* copies on the chromosome (a possible total of 21 copies per genome). Possible OC8 mutants with larger deletions may also have been eliminated during evolution.

Although IS*256* has extensively been investigated [27,30,33,34,48,49,65], precise analysis of cell-to-cell spread and genome-wide/intracellular distribution of IS*256* has not been reported before. Regarding the cell-to-cell spread of IS*256*, we speculate that one copy of IS*256* was introduced into OC8 (its ancestral strain). We confirmed the presence of an IS*256* extrachromosomal circular DNA in OC8, as reported previously [48,49]. It is conceivable that the circular form of IS*256* is transferred from cell to cell, similar to the erythromycin resistance transposon Tn*554* (in ST239/SCC*mec*III HA-MRSA), which formed a circular DNA [41,66] and was successfully transferred by conjugation (in bacterial mixed cultures), as a “transmissible transposon” [41,42]. We clearly demonstrated that small plasmids, such as 2.9-kb chloramphenicol resistance plasmids, are transferred in *S*. *aureus* by conjugation (in bacterial mixed cultures) at a markedly higher frequency than “transmissible” large penicillinase (PCase) plasmids [41,42]. This mode of inter-bacterial transmission may also strongly stimulate the spread of IS*256* among *S*. *aureus*. The circular form of IS*256* is now being investigated to verify its cell-to-cell transfer (as a “transmissible insertion sequence”).

Regarding the inner-cellular spread of IS*256*, the behavior of IS*256* was flexible. i) Although the notion that IS*256* insertions may not occur randomly has been reported previously [34], we found three IS*256*-enriched recombination hot spots on the OC8 genome, in addition to a series of the single location of IS*256*. The molecular mechanisms underlying this gathered manner of the IS*256* insertion have not yet been elucidated. However, hot spots may reflect gathered IS*256*-preferred insertion site sequences, may occur at “junk” regions on the genome, or hot spot regions may provide a unique topological circumstance that boosts the attack of IS*256* transposase. ii) IS*256*-flanking *att* sequences (8 or 9 pb in size) detected on the OC8 genome were all divergent, suggests that *att* site selection is not strict (frequency of the appearance of the same *att* sequence, <5.3% [<1/19] or <4.8% [<1/21]). IS*256* extrachromosomal circular DNA also existed in various forms, each with a 6-bp heterogeneous stretch. Therefore, the IS*256* transposase, which is a DNA-binding protein (49), preferred sizes rather than fixed unique sequences. iii) IS*256*-flanking *att* sequences exhibited three distinct flanking manners: homogenous *att*L and *att*R as DR (73.7%), which are created upon insertions; heterogeneous *att*L and *att*R at hot spots (10.5%), which are created upon inversions or deletions, subsequent to insertions; and partially homologous *att*L and *att*R as imperfect flanking DR (15.8%), which are made upon insertions and subsequent *att*R mutation(s) through unknown mechanism(s). In the present study, therefore, heterogeneous *att*L and *att*R indicated the presence of inversions or deletions.

Regarding the role of IS*256* in evolution, in addition to the above chromosomal rearrangements, we emphasize the successful spread of IS*256* among MRSA in Russia. The prevalent ST8/SCC*mec*IVc CA-MRSA lineage (strain OC8), prevalent ST239/*spa*3(t037)/SCC*mec*IIIA HA-MRSA lineage (strain OC3) [42], and prevalent ST239/*spa*351(t030)/SCC*mec*III_R_ HA-MRSA lineage (strain 16K) [41] all carried multiple IS*256* copies, strongly suggests that an IS*256* multicopy system confers a selective advantage on its host and boosts MRSA evolution.

Regarding genome sequencing technology and an IS*256* analysis, in our previous comparative genomic analyses, on OC3 [42] and 16K [41], we used pyrosequencing technology without filling all the gaps between contigs (except for relevant genetic structures); therefore, a precise analysis of IS*256*-adjacent sequences or the large inversion was not performed. In the present study, since we used PacBio RS II system technology [46] and also completed making a complete circular genome sequence, we succeeded in conducting precise analysis on genome-wide IS*256* distribution and the large genomic inversion. A small plasmid analysis was not available for the PacBio RS II system, because small DNA pieces, less than approximately 20 kb, were removed, and only large DNA pieces were employed for library construction. Therefore, for small plasmids, we isolated plasmid DNA in separate experiments for a complete plasmid sequence analysis using previously described methods.

Regarding the effects of IS*256* on gene expression, IS*256* has been considered to alter *S*. *aureus* virulence and drug resistance [30]. For example, the insertion of IS*256* into the *rot* promoter has been shown to affect virulence levels [30]. In the present study, an IS*256* insertion was not observed in the *rot* promotor region [30] or *ica* regions [34,65]. Regarding genomic islands, vSAα, vSAβ, and vSAγ all had an IS*256* insertion. Of these, vSAβ was split into two parts by an MbIN event, with a small deletion. The possible association of IS*256* insertions, MbIN, and deletions with virulence expression is under investigation.

Regarding the global geographical structures of MRSA, several continental clades and intercontinental spread have been reported for the ST239/SCC*mec*III HA-MRSA lineage [39], albeit with no Russian isolates. Heterogeneous ST239/SCC*mec*III sub-lineages are distributed in Russia [38], such as emerging ST239/*spa*351(t030)/SCC*mec*III_R_ in Vladivostok [41] and ST239/*spa*3(t037)/SCC*mec*IIIA (ST239_Kras_) in Krasnoyarsk [42]. The latter, ST239_Kras_, represented the Siberian Russian clade of the ST239 HA-MRSA lineage, with the possible evolutionary routes of Brazil-Europe-Siberian Russia [42].

In the case of the globally disseminated ST8/SCC*mec*IV CA-MRSA lineage, USA300 (ST8/*spa*1[t008]/SCC*mec*IVa with PVL-encoding φSa2 and ACME linked to SCC*mec*IVa) is the most successful example [2,4,9–12,14,15]. USA300 caused the largest MRSA epidemic in the United States [4,11,12,14,32], exhibited intercontinental transmission [8,32,67], and has been the most precisely characterized among MRSA [4,9–12,14,15,37]. Many other geographical variants of the ST8/SCC*mec*IV CA-MRSA lineage have also been described [68]. We previously reported prevalent CA-MRSA with ST8/*spa*606(t1767)/SCC*mec*IVl (ST8 CA-MRSA/J) in Japan, which carried a unique Japanese subtype of SCC*mec*IV (SCC*mec*IVl) and novel SaPIj50 with the *tst* gene [69]; and also ST8 CA-MRSA with *spa*779(tUK: 11-19-12-21-17-34-24-24-34-24-24-34-22-25)/SCC*mec*IVx (subtype unknown) in Taiwan; however, the prevalence of ST8 MRSA in Taiwan was low (2.4%) [20]. In Asia, SCC*mec* types of the ST8 lineage may be highly divergent and unique.

In the present study, based on the OC8 complete genome data, we established the Russian clade of the ST8/SCC*mec*IV CA-MRSA lineage. Russia ST8-IVc had a genetic marker of unique MbIN (triggered by IS*256* hot spots), spread widely to European, Siberian, and Far Eastern Russia with geographical microevolution, including *spa* types, and was associated with not only SSTIs, but also serious and invasive infections, such as pneumonia, sepsis, and bloodstream infections, in both community and hospital settings [this study, 41,42]. The global evolutionary route of Russia ST8-IVc remains to be elucidated.

Regarding multiple drug resistance (MDR), Russia ST8-IVc strains from European and Siberian regions exhibited levofloxacin (ciprofloxacin) resistance, providing a selective advantage for Russian ST8-IVc, similar to USA300 cases [11,32,67]. Common chloramphenicol resistance also appears to provide a selective advantage for Russia ST8-IVc because chloramphenicol is one of the most common drugs used in chemotherapy in Russia [42]. Rifampicin resistance may have been selected due to tuberculosis treatments [70] or by the geographical common use of rifampicin.

Concerning multiple virulence factors (MVFs) of MRSA, although PVL genes and ACME were not present, unlike USA300 [15], Russia ST8-IVc (including OC8) carried, for example, the SAg-SEA gene (*sea*), strongly expressed *psmα*, *hla*, and a series of immune evasion genes, such as *spa*, *ebh*, *map*, *scn*, *sak*, *sbi*, *fnbA*, and *fnbB*, which have been reported previously [4,8,9,10,12,30,42,71]. Although we have not fully analyzed gene mutations in OC8, the very large gene *ebh* (encoding for the giant protein Ebh [52,53]) had a premature stop codon; therefore its product, truncated Ebh (Ebh∆), was predicted to possess the N-terminal signal peptide, FIVAR repeats, and a part of the extension of FIVAR/GA modules, but lacked the bulk of the extension of FIVAR/GA modules, the transmembrane domain, and C-terminal-positive charges [52,53], thereby losing its function as a very large surface-anchored protein. The nonsense mutation in the *ebh* gene was unique to strain OC8 (ST8_Kras_). Other ST8_Kras_ strains (OC11 and OC22), Russia ST8-IVc strains from St. Petersburg and Vladivostok (S214 and 12K), and ST239/SCC*mec*III strains (OC3 and 16K) did not have the OC8-type nonsense mutation in the *ebh* gene; this point is further under investigation.

One IS*256* insertion occurred 521-bp upstream of *rsp* (the gene for the AraC family transcriptional regulator [72]), suggesting an influence on the regulation system for virulence genes. In order to gain a more precise understanding of the gene expression and potential virulence of Russia ST8-IVc (OC8), further investigations are needed.

Regarding the ST8/SCC*mec*IV CA-MRSA lineage, factors associated with a successful clonal expansion in each region/country include i) MDR, not only resistance to globally important agents (e.g., fluoroquinolones) but also resistance to regionally common agents (e.g., chloramphenicol), ii) the ability of powerful adherence, colonization, and spread, and iii) enhanced MVFs.

In conclusion, we determined the complete circular genome sequence of ST8/*spa*1(t008)/SCC*mec*IVc CA-MRSA (ST8_Kras_ strain OC8). This enabled us to gain novel insights into the following. i) Regarding large genomic rearrangements, OC8 had MbIN, the largest genomic inversion in MRSA, and vSAβ (OC8) essentially split. Its impact is unknown, however, since MbIN was a common feature of successful Russian ST8-IVc, it was not a fitness burden. MbIN was unambiguously diagnosed by PCR. ii) Regarding IS*256*’s spread and functions, it was of special interest that the ST8 CA- and ST239 HA-MRSA lineages in Russia all carried multi-IS*256*. We speculate that IS*256* has strong transmission potential and epidemiological advantages. IS*256* exhibited flexible manners at the integration stage and extrachromosomal DNA stage, and acted as a powerful trigger for MRSA evolution, for example, IS*256* at its hot spots created MbIN. iii) Regarding MVFs, we found additional virulence factors of OC8, such as the truncated giant surface protein Ebh∆ and IS*256* insertion related to pan-regulation. iv) Regarding global geographical structures, we assigned Russia ST8-IVc as a new powerful clade of the globally disseminated ST8/SCC*mec*IV CA-MRSA lineage. Russia ST8-IVc was geographically expanded in both community and hospital settings since approximately 2006, with characteristic MbIN as an epidemiological marker and fluoroquinolone resistance, increased MVFs, and possibly a multi-IS*256* system as selective advantages. The evolutionary route of Russia ST8-IVc remains to be elucidated.

## Supporting Information

S1 Fig Structure of φSa3 in strain OC8. φSa3 (OC8) exhibited the highest homology to φSa3 (T0103). The left-side immune evasion cluster (IEC) region of φSa3 (OC8) also exhibited high homology to φSa3 (TW20). Homologous regions are shaded in each comparison. Genes in IEC: *scn*, the staphylococcal complement inhibitor (SCIN) gene; *sak*, the staphylokinase (SAK) gene; *sea*, the staphylococcal enterotoxin A (ETA) gene. OC8 lacked *chp*, the chemotaxis inhibitory protein of the *S*. *aureus* (CHIPS) gene, unlike USA300 FPR3757 (GenBank accession number CP000255).
(TIF)

S2 Fig Comparison of the *ebh* gene and its product between OC8 and USA300 FPR3757. The nucleotide sequence of *ebh* and deduced amino acid sequence of Ebh were compared between USA300 FPR3757 (upper side) and OC8 (lower side) in A and B, respectively. In A, *ebh* (OC8) had three synonymous substitutions (black), two non-synonymous substitutions (red), and one nonsense mutation (blue). In order to confirm the nonsense mutation, we designed two primer sets, ebh1F and ebh1R (5'-GTGTTCAAACGGTTCAATCA and 5'-AATAATCGTTTCAGCAGCAG, generating a 170-bp product) and ebh2F and ebh2R (5'-ACTTAGATGGTACGCGTTTA and 5'-AACTATTCACTTGCTCTGCT, generating a 369-bp product) based on the OC8 genome (*ebh*) sequence. The PCR with those primers and OC8 DNA and subsequent sequencing perfectly confirmed the nonsense mutation (G→T at position 11,029 bp). Due to the nonsense mutation at B, the *orf* of *ebh* (OC8) was shortened, and corresponded to only 35.3% of *ebh* (USA300 FPR3757). In B, Ebh (USA300 FPR3757) was 10,421 aa in length, while truncated Ebh (OC8) was only 3, 676 aa, corresponding to 35.3% of Ebh (USA300 FPR3757). Truncated Ebh (OC8), Ebh∆, showed 100% homology to the corresponding region of Ebh (USA300 FPR3757), but lacked the bulk of FIVAR GA modules and transmembrane domain of Ebh [52,53].
(TIF)

S3 Fig Possible mechanism for a deletion at the IS*256*⑪ site in OC8. In this model, we hypothesized ancestor strains of OC8 for a deletion event. An initial ancestor strain (OC8 ancestor 1) lacks IS*256*, but has *att* site sequences, similar to USA300 FPR3757; the size of OC8 ancestor 1 DNA flanked by two *att* sites was estimated to be 63 bp. The first step (step 1) includes two IS*256* insertions at different *att* sites (generating OC8 ancestor 2). In step 2, a homogenous recombination occurs between direct repeats of IS*256* (in OC8 ancestor 2), deleting a small region and leaving only one copy of IS*256* (generating OC8 with IS*256*⑪).
(TIF)

S4 Fig The structure of IS*256* extrachromosomal circular DNA in the ST239/SCC*mec*III HA-MRSA lineage in Russia. In A, the structure of IS*256* (OC8) is based on the OC8 genome sequence (GenBank accession number AP017377) and is the same as that described in Fig 4A. In A and B, strains OC3 and 16K were examined for the circle junction of IS*256* circular molecules by PCR, using PCR primer set (R-R1 and L-R2). Their amplified bands were very similar to that of OC8. In C, the sequence of the PCR products, estimated, perfectly matched the IR_L_ side and IR_R_ side regions of IS*256* (OC8), and contained the 6-bp stretch, marked in red; 26-bp imperfect IR sequences and 6-bp stretch sequences are underlined. The 6-bp stretch data showed a “mixed” result, with TTGTGT (for 16K) or TATTTT (for OC3) as a highest base content, most probably reflecting divergent *att* sequences on each genome.
(TIF)
