# Supplementary figures and images for: Complete Circular Genome Sequence of Successful ST8/SCCmecIV Community-Associated Methicillin-Resistant Staphylococcus aureus (OC8) in Russia: One-Megabase Genomic Inversion, IS256’s Spread, and Evolution of Russia ST8-IV
Source: PLoS One. 2016 Oct 14;11(10):e0164168. doi: 10.1371/journal.pone.0164168 (PMC5065196; doi:10.1371/journal.pone.0164168)

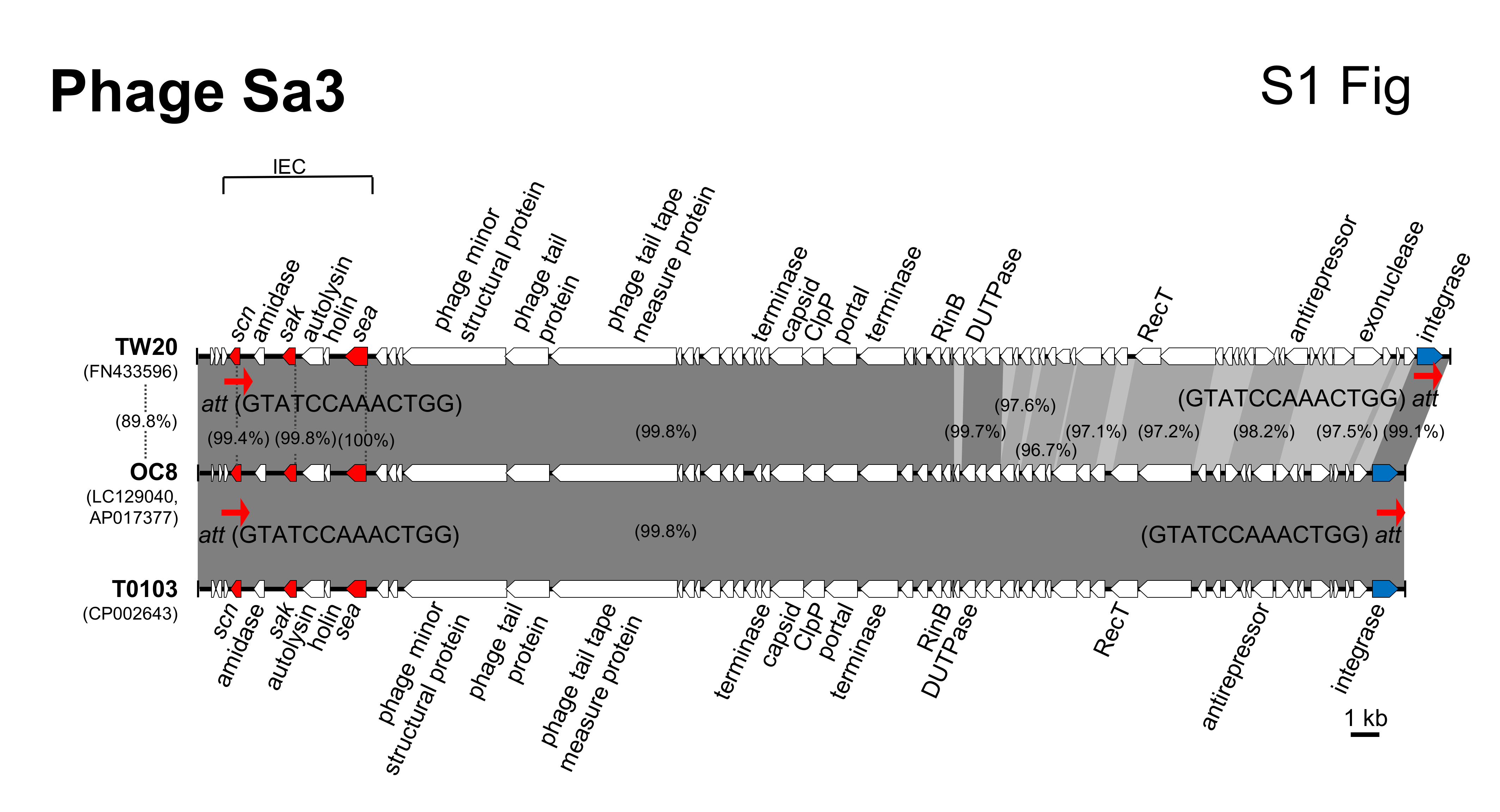

Supplement: S1 Fig — φSa3 (OC8) exhibited the highest homology to φSa3 (T0103). The left-side immune evasion cluster (IEC) region of φSa3 (OC8) also exhibited high homology to φSa3 (TW20). Homologous regions are shaded in each comparison. Genes in IEC: scn, the staphylococcal complement inhibitor (SCIN) gene; sak, the staphylokinase (SAK) gene; sea, the staphylococcal enterotoxin A (ETA) gene. OC8 lacked chp, the chemotaxis inhibitory protein of the S. aureus (CHIPS) gene, unlike USA300 FPR3757 (GenBank accession number CP000255). (TIF) [file pone.0164168.s001.tif]

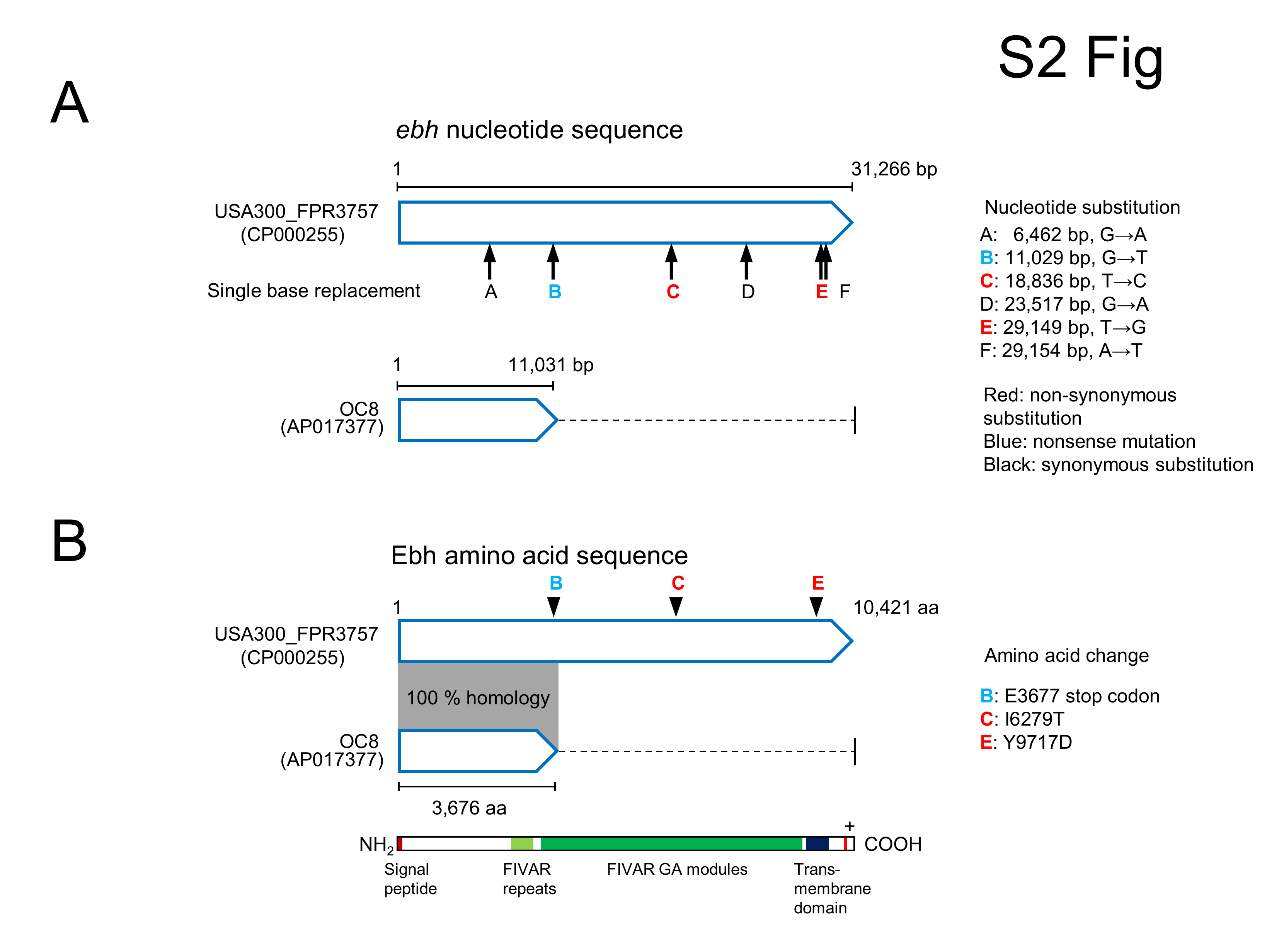

Supplement: S2 Fig — The nucleotide sequence of ebh and deduced amino acid sequence of Ebh were compared between USA300 FPR3757 (upper side) and OC8 (lower side) in A and B, respectively. In A, ebh (OC8) had three synonymous substitutions (black), two non-synonymous substitutions (red), and one nonsense mutation (blue). In order to confirm the nonsense mutation, we designed two primer sets, ebh1F and ebh1R (5'-GTGTTCAAACGGTTCAATCA and 5'-AATAATCGTTTCAGCAGCAG, generating a 170-bp product) and ebh2F and ebh2R (5'-ACTTAGATGGTACGCGTTTA and 5'-AACTATTCACTTGCTCTGCT, generating a 369-bp product) based on the OC8 genome (ebh) sequence. The PCR with those primers and OC8 DNA and subsequent sequencing perfectly confirmed the nonsense mutation (G→T at position 11,029 bp). Due to the nonsense mutation at B, the orf of ebh (OC8) was shortened, and corresponded to only 35.3% of ebh (USA300 FPR3757). In B, Ebh (USA300 FPR3757) was 10,421 aa in length, while truncated Ebh (OC8) was only 3, 676 aa, corresponding to 35.3% of Ebh (USA300 FPR3757). Truncated Ebh (OC8), Ebh∆, showed 100% homology to the corresponding region of Ebh (USA300 FPR3757), but lacked the bulk of FIVAR GA modules and transmembrane domain of Ebh [52,53]. (TIF) [file pone.0164168.s002.tif]

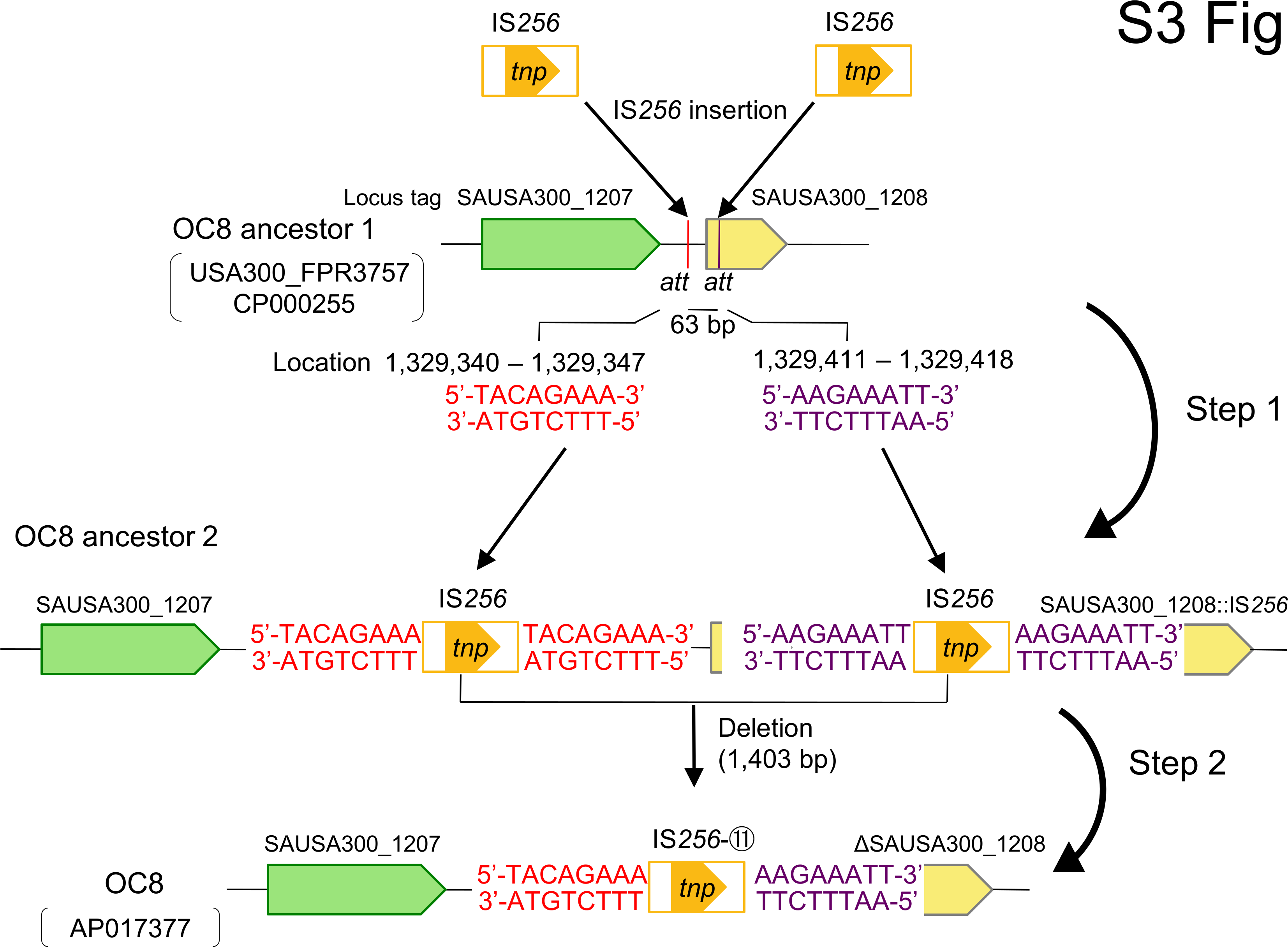

Supplement: S3 Fig — In this model, we hypothesized ancestor strains of OC8 for a deletion event. An initial ancestor strain (OC8 ancestor 1) lacks IS256, but has att site sequences, similar to USA300 FPR3757; the size of OC8 ancestor 1 DNA flanked by two att sites was estimated to be 63 bp. The first step (step 1) includes two IS256 insertions at different att sites (generating OC8 ancestor 2). In step 2, a homogenous recombination occurs between direct repeats of IS256 (in OC8 ancestor 2), deleting a small region and leaving only one copy of IS256 (generating OC8 with IS256⑪). (TIF) [file pone.0164168.s003.tif]

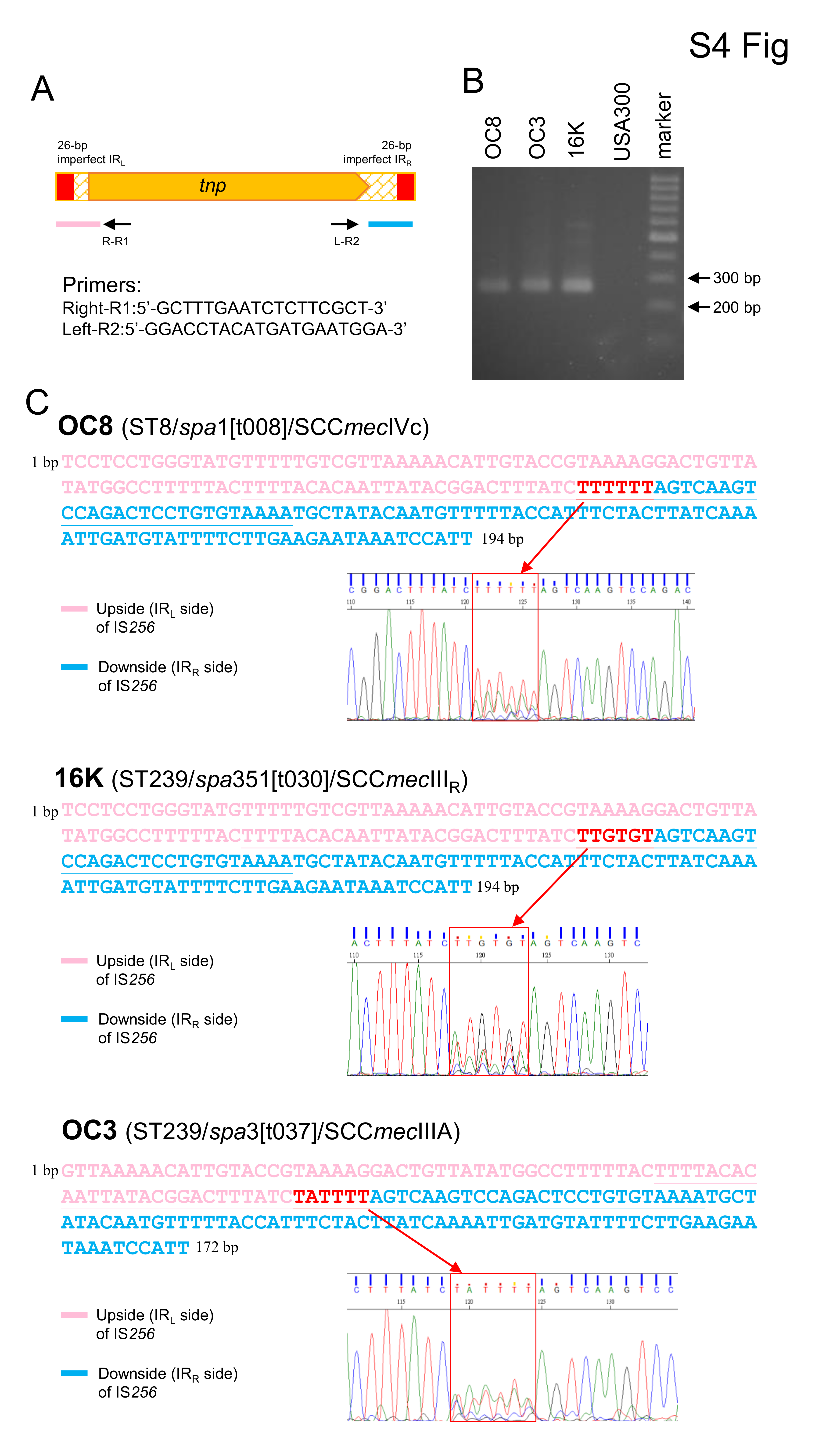

Supplement: S4 Fig — In A, the structure of IS256 (OC8) is based on the OC8 genome sequence (GenBank accession number AP017377) and is the same as that described in Fig 4A. In A and B, strains OC3 and 16K were examined for the circle junction of IS256 circular molecules by PCR, using PCR primer set (R-R1 and L-R2). Their amplified bands were very similar to that of OC8. In C, the sequence of the PCR products, estimated, perfectly matched the IRL side and IRR side regions of IS256 (OC8), and contained the 6-bp stretch, marked in red; 26-bp imperfect IR sequences and 6-bp stretch sequences are underlined. The 6-bp stretch data showed a “mixed” result, with TTGTGT (for 16K) or TATTTT (for OC3) as a highest base content, most probably reflecting divergent att sequences on each genome. (TIF) [file pone.0164168.s004.tif]
